# Effects of extrapulmonary TB on patient quality of life and recurrence

**DOI:** 10.5588/pha.24.0012

**Published:** 2024-09-01

**Authors:** A. Wali, N. Safdar, A. Ambreen, S. Hassan, A. Yaqoob, T. Mustafa

**Affiliations:** ^1^ Centre for International Health, Department of Global Public Health and Primary Care, University of Bergen, Bergen, Norway;; ^2^ Department of Health, Government of Balochistan, Quetta, Pakistan;; ^3^ Interactive research and development, Singapore, Singapore;; ^4^ Department of Microbiology, Gulab Devi Hospital, Lahore, Pakistan;; ^5^ Common Management Unit (HIV/AIDS, TB & Malaria), Islamabad, Pakistan;; ^6^ Department of Thoracic Medicine, Haukeland University Hospital, Bergen, Norway.

**Keywords:** pleuritis, lymphadenitis, health-related, Pakistan

## Abstract

**BACKGROUND:**

Quantifying quality of life (QoL) in extrapulmonary TB patients is crucial yet often overlooked. This study examines the impact of tuberculous lymphadenitis and pleuritis on patients' QoL, associated factors, and recurrence.

**METHODS:**

Data were collected prospectively from patients using a pre-designed questionnaire at baseline, post-treatment, and 2 years after treatment. Health domains are essential to overall health and well-being and can be assessed to understand health status. We included mobility for physical well-being, usual activities for self-care, pain/discomfort for disease manifestations, and anxiety/depression for emotional health.

**RESULTS:**

Of the 376 patients, 53% had TB lymphadenitis, and 47% had TB pleuritis, with a mean age of 25 years (SD ±12.95). The most commonly reported issues at baseline were pain/discomfort and restricted usual activities. After treatment, over 90% experienced improvement, but 8% re-developed symptoms after 2 years, and two patients required retreatment for recurrent EPTB. Predictors negatively impacting QoL included private income sources, residence outside the city, and marriage, with the first two primarily affecting emotional health.

**CONCLUSION:**

Tuberculous lymphadenitis and pleuritis significantly impact patients' physical and emotional health, necessitating healthcare providers to address non-medical factors affecting QoL to reduce morbidity and mortality and improve QoL.

## Abstract (fr)

**CONTEXTE:**

La quantification de la qualité de vie (QoL) chez les patients atteints de TB extrapulmonaire (EPTB) est essentielle mais fréquemment ignorée. Cette recherche analyse l'influence de la lymphadénite et de la pleurésie tuberculeuse sur la QoL des patients, les facteurs associés et la récurrence.

**MÉTHODES:**

Les données ont été collectées de manière prospective auprès des patients à l'aide d'un questionnaire préétabli, initialement, post-traitement et deux ans après le traitement. Les aspects de la santé sont fondamentaux pour le bien-être général et peuvent être évalués afin de comprendre l'état de santé. La mobilité a été incluse pour le bien-être physique, les activités quotidiennes pour les soins personnels, la douleur/l'inconfort pour les symptômes de la maladie et l'anxiété/la dépression pour la santé émotionnelle.

**RÉSULTATS:**

Sur les 376 patients, 53% ont été diagnostiqués avec une lymphadénite tuberculeuse et 47% avec une pleurite tuberculeuse, avec un âge moyen de 25 ans (SD ±12,95). Les symptômes les plus couramment signalés initialement étaient la douleur/l'inconfort et la limitation des activités habituelles. Après le traitement, plus de 90% des patients ont constaté une amélioration de leur état, mais 8% d'entre eux ont présenté à nouveau des symptômes après deux ans, et deux patients ont nécessité un nouveau traitement pour une EPTB récurrente. Les facteurs prédictifs qui ont un effet négatif sur la QoL incluaient les sources de revenus privées, la résidence en dehors de la ville et le mariage, les deux premiers ayant principalement un impact sur la santé émotionnelle.

**CONCLUSION:**

La lymphadénite tuberculeuse et la pleurite ont un impact important sur la santé physique et émotionnelle des patients, ce qui nécessite que les professionnels de la santé abordent les facteurs non médicaux qui affectent la QoL afin de diminuer la morbidité et la mortalité, et d'améliorer la QoL.

Extrapulmonary TB (EPTB) is a significant clinical expression of TB, accounting for up to 17% of all newly diagnosed and recurrently reported cases globally in 2022.^1^ Tuberculous lymphadenitis (TBLN) and tuberculous pleuritis (TBP) are the most common manifestations, constituting approximately 50–55% of all EPTB cases.^2–4^ Compared to pulmonary TB (PTB), EPTB has received less priority in public health programs due to its non-contagious nature, leaving many cases undetected. The diverse clinical presentations and paucibacillary nature of the disease pose significant diagnostic challenges, further complicated by delays in seeking healthcare due to complicated pathways and the poor socioeconomic status of patients. This leads to disease progression, complications, and adverse health outcomes, resulting in unfavourable treatment outcomes and increased recurrence rates.^5–7^ These factors significantly impact patients' quality of life (QoL) and their perceptions of well-being.

The aim of this study was to investigate the impact of TBLN and TBP on patient-reported quality of life (PR-QoL), associated factors, short-term and long-term treatment effects, and recurrence among EPTB patients at a tertiary care hospital in Lahore, Pakistan, as there is limited evidence on their physical and psychological effects.^8–10^

## METHODS

This study is part of a larger research project evaluating the efficacy of a new diagnostic MPT64 antigen detection test for EPTB.^11^ It used a prospective cohort design, assessing common health domains such as mobility, usual activities, pain/discomfort, and anxiety/depression to evaluate the health-related QoL of patients with TBLN and TBP in Pakistan. The impact of treatment on PR-QoL at the end of treatment and the recurrence of QoL problems or disease symptoms after 2 years were the outcomes of interest.

### Study setting and population

The study was conducted at Gulab Devi, a not-for-profit private tertiary care hospital in Lahore, Pakistan, engaged with the National TB Control Programme. Patients aged ≥10 years presumed to have TBLN or TBP were investigated, diagnosed, and enrolled in anti-TB treatment (ATT) at outpatient department clinics from April 2016 to August 2017 and followed up. Patients without written consent or those who had received ATT in the previous year were excluded.

### Study questionnaire

A questionnaire adapted from a multicentre study, with slight modifications,^11^ was used. It was translated into Urdu and back-translated to English to ensure validity. The questionnaire covered various sections, assessing PR-QoL using four health measurement domains: mobility, usual activities, pain/discomfort, and anxiety/depression.^8^

### Data collection

Data on demographic, socioeconomic, healthcare-seeking, and EPTB disease-specific symptoms and signs and clinical evaluations were collected at baseline. PR-QoL data were gathered through face-to-face interviews at enrolment and via telephone at the end of treatment and 2 years post-treatment, including the redevelopment of symptoms and disease recurrence. Children aged 10–14 years were supported by a guardian during data collection.

### Data analysis and statistics

SPSS v20.0.0 (IBM Corp, Armonk, NY, USA) was used for statistical analyses. Proportions of respondents were calculated, and χ^2^ tests determined the statistical significance of differences across groups. Direct logistic regression analysis explored factors affecting patients' QoL and health, with categorical outcomes coded as 0 for no problem and 1 for some or extreme problems. *P* < 0.05 was considered statistically significant.

### Ethics approval and consent to participate

Ethical clearance was obtained from the Regional Committee for Medical and Health Research Ethics, Western Norway, and the National Bioethics Committee of Pakistan. Study participants signed informed written consent forms, with parental consent for participants aged 10–14 years.

## RESULTS

**FIGURE 1.**
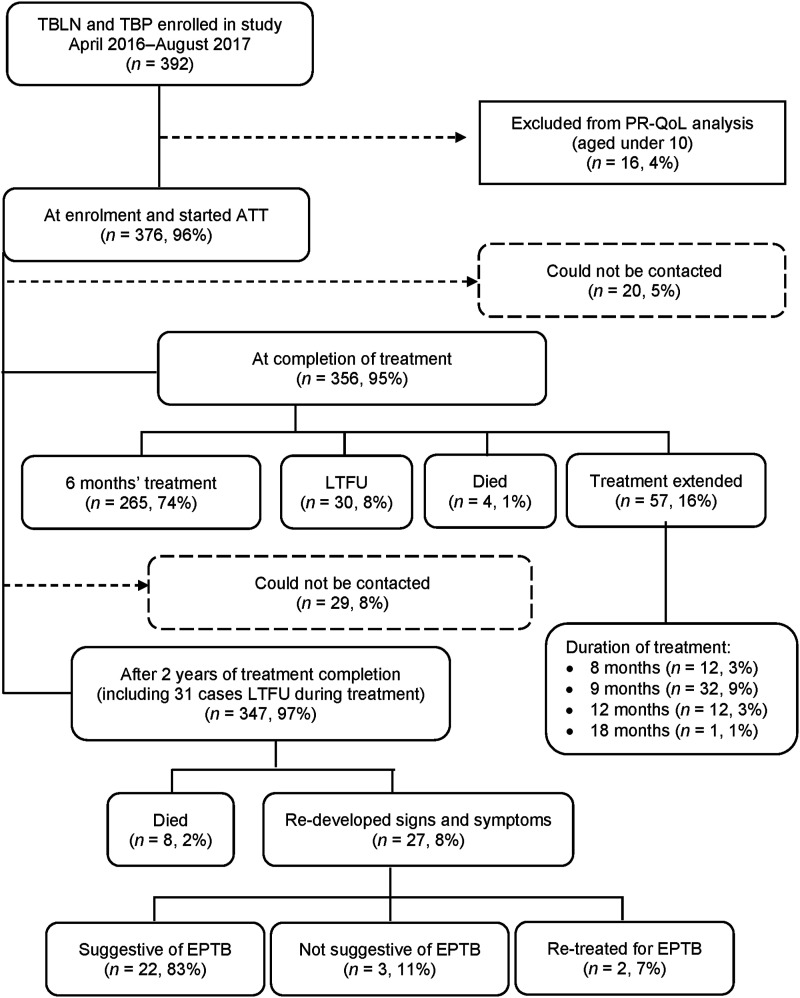
Study profile with short- and long-term outcomes. The boxes contain the study population; the solid lines show the number of patients included, and the dotted lines indicate patient exclusion based on the exclusion criteria. TBLN = tuberculous lymphadenitis; TBP = tuberculous pleuritis; PR-QOL = patient-reported quality of life; ATT = anti-TB treatment; LTFU = loss to follow-up; EPTB = extrapulmonary TB.

Figure 1 shows that 376 patients were analysed. Among the 356 follow-up patients at treatment completion, 74% had received ATT for 6 months, while 16% required treatment extensions up to 6–18 months due to persistent symptoms. Four patients (1%) died, and 30 (8%) were lost to follow-up. Most extensions (82%) were for TBLN patients. At treatment end, 29 (8%) patients could not be contacted for follow-up.

Table 1 details the sociodemographic, economic, and clinical characteristics of 392 patients with TBLN and TBP. Of these, 52% had TBLN, while 48% had TBP. The majority (60%) were aged ≥26 years, 52% were male, 55% were unmarried, and 45% had completed primary education; 71% relied on private employment salaries, while 8% were engaged in agriculture. Around 57% sought healthcare within 2 weeks, and 62% were diagnosed within ≥5 weeks.

**TABLE 1.** Baseline characteristics of the study participants.

Characteristics		At baseline (*n* = 376)
		*n (*%)
Age, years	10–24	214 (56.9)
	25–44	121 (32.2)
	>45	41 (10.9)
Sex	Male	194 (51.6)
	Female	182 (48.4)
Marital status	Single	207 (55.1)
	Married	169 (44.9)
Education levels*	Primary school	170 (45.2)
	Secondary education	58 (15.4)
	Higher secondary	28 (7.4)
	Higher education	15 (4.0)
	Adult education^†^	7 (1.9)
	No formal education	98 (26.1)
Source of income	Agriculture	30 (8.0)
	Government salary	25 (6.6)
	Private sector	268 (71.3)
	Self-employed	39 (10.4)
	Other^‡^	14 (3.7)
Household monthly income, PKR	<10,000	53 (14.1)
	10,000–30,000	307 (81.6)
	31,000–50,000	16 (4.3)
Household size (number of members)	1–5	55 (14.6)
	6–10	258 (68.6)
	>11	63 (16.8)
Working capacity	Working normal	124 (33.0)
	Reduced	222 (58.0)
	Completely stopped	30 (8.0)
Sites of EPTB infection	TB lymphadenitis	195 (51.9)
	TB pleuritis	181 (48.1)
Time to seek healthcare, weeks	1–2	213 (56.6)
	2–4	53 (14.1)
	>5	110 (29.3)
Time to diagnosis, weeks	1–2	77 (20.5)
	2–4	67 (17.8)
	>5	232 (61.7)

* Levels of education frame work in Pakistan, 2015.

† Full formal education of adults.

‡ Housewives and student participants.

EPTB = extrapulmonary TB; PKR = Pakistani rupee.

**FIGURE 2.**
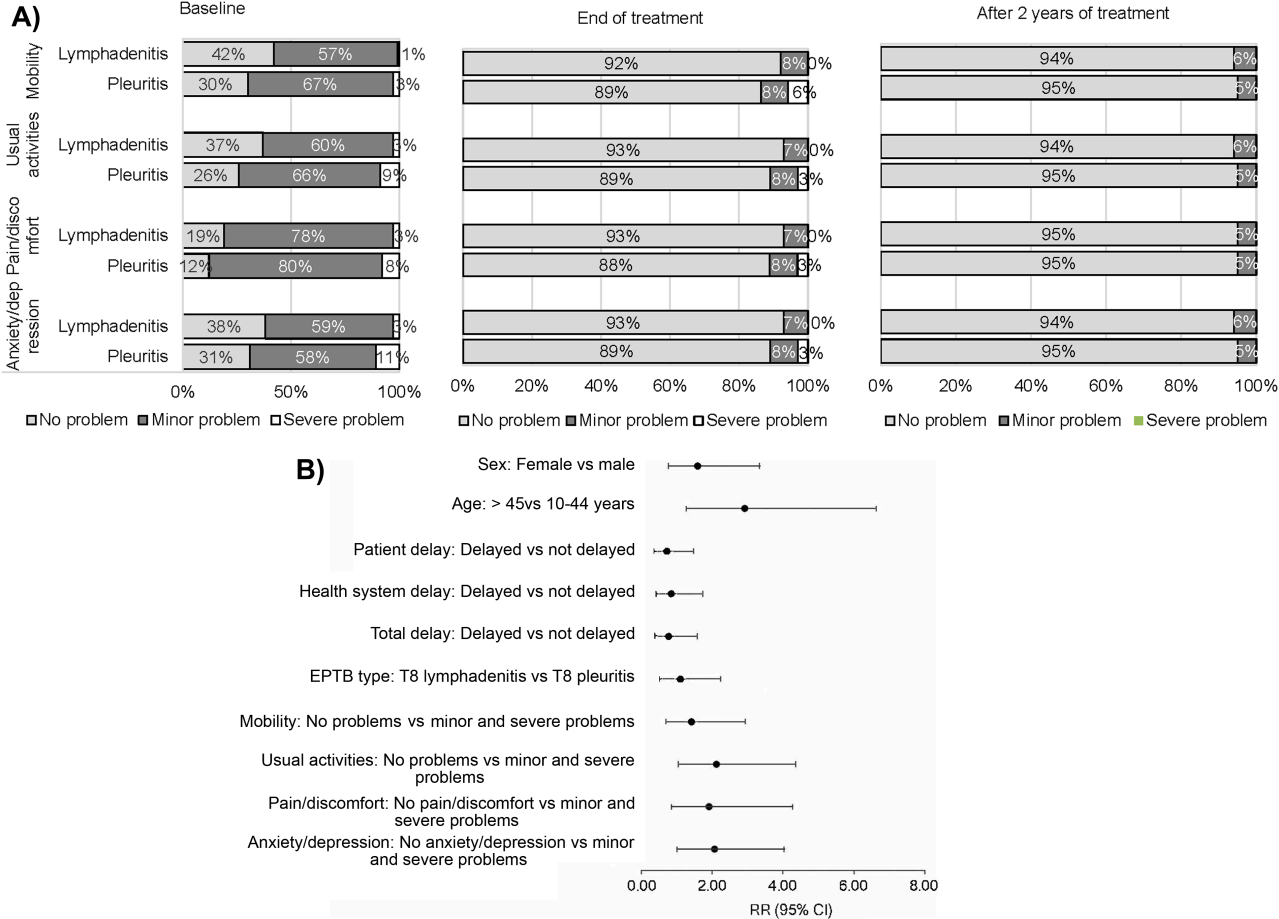
Patient statements and effect estimates of risk factors on problem recurrence. **A)** Stacked bar plot shows the proportion of TB lymphadenitis and TB pleuritis patients reporting the quality of life in each of the four health domains: at baseline, at the end of anti-TB treatment, and after 2 years of treatment completion. At baseline, approximately 58–88% of the patients experienced some or extreme problems in all four health-related domains, which decreased to only 9% after completion of treatment. At the end of treatment, TB lymphadenitis patients showed a slightly greater improvement than TB pleuritis patients. Reported problems decreased after 2 years of treatment. **B)** Forest plot showing the relative risk of recurrence of the problems among 27 TB lymphadenitis and TB pleuritis patients after 2 years of treatment completion; closed circles indicate means and whiskers represents the 95% CIs. RR = relative risk; CI = confidence interval; EPTB = extrapulmonary TB.

Over 90% of patients experienced improvements after treatment, with pain or discomfort and usual activities being the most significant health issues (Figure 2A). Before treatment, 64%, 68%, 84%, and 65% of patients reported some or extreme problems with mobility, usual activities, pain or discomfort, and anxiety or depression. Post-treatment, only 9% reported extreme or some issues in all four health domains. TBLN patients reported slightly greater improvement compared to TBP patients.

Table 2 shows problems reported by respondents in four health domains at baseline. The most common issues were pain or discomfort (84%), followed by restriction of usual activities (68%) and mobility (64%). These problems were more prevalent among male respondents (51%), those with reduced working capacity (71%), and TBP patients (70%). Individuals living outside the city and those with reduced working capacity faced difficulties with usual activities, and TBLN patients experienced more healthcare delays and pain or discomfort. Younger respondents (10–24 years) experienced more anxiety or depression (51%), especially those married, living outside the city, and working for the government. Additionally, 75% of patients who died experienced delays in seeking healthcare, and 33% experienced diagnostic delays.

**TABLE 2.** Proportion of patients reported any problem in four health domains at baseline.

Characteristic	Mobility			Usual activities			Pain/discomfort			Anxiety/depression		
	No problems	Minor and severe problems	*P* value	No problems	Minor and severe problems	*P* value	No problems	Minor and severe problems	*P* value	No problems	Minor and severe problems	*P* value
	%	%		%	%		%	%		%	%	
Total	37	64		32	68		16	84		35	65	
Age, years												
10–14	46	54	0.20	42	59	0.15	19	82	0.73	49	51	0.01
15–64	35	65		30	70		15	85		31	69	
≥65	33	67		22	78		11	89		44	56	
Sex												
Male	37	63	0.82	32	68	0.87	14	86	0.38	39	61	0.06
Female	36	64		31	69		17	83		30	70	
Residence												
Urban	40	60	0.06	34	66	0.26	20	80	0.00	38	62	0.05
Rural	31	69		28	72		09	91		29	71	
Marital status												
Single	39	61	0.32	36	64	0.02	18	82	0.13	40	60	0.00
Married	34	66		25	75		12	88		27	73	
Educational level												
Basic elementary	41	59	0.07	37	63	0.00	17	83	0.51	39	61	0.08
Secondary	38	62		36	64		12	88		38	62	
Higher secondary	46	54		46	54		18	82		43	57	
Higher education	40	60		20	80		27	73		40	60	
Adult education	14	86		00	100		00	100		14	86	
No formal education	25	75		18	82		13	87		23	76	
Source of income												
Agriculture	44	56	0.12	38	63	0.05	13	88	0.06	34	66	0.04
Government salary	48	52		41	59		15	85		30	70	
Private sector	33	67		28	73		13	87		32	69	
Self-employed	42	58		40	61		26	74		51	49	
Other	57	43		57	43		36	64		57	43	
Monthly income, PKR												
<10,000	38	62	0.96	26	74	0.23	12	88	0.35	38	62	0.78
10,000–30,000	36	64		33	67		17	83		34	66	
31,000–50,000	35	65		18	82		06	94		29	71	
Household size												
1–5	35	65	0.96	37	63	0.64	19	81	0.60	42	58	0.47
6–10	37	64		31	69		14	86		33	67	
>11	38	63		30	70		17	83		34	66	
Working capacity												
Working normal	48	52	0.00	46	54	0.00	21	79	0.07	48	52	0.00
Reduced	29	71		23	77		12	88		26	74	
Completely stopped	43	57		27	73		17	83		37	63	
EPTB infection sites												
TBP	30	70	0.01	26	74	0.01	12	88	0.07	31	69	0.16
TBLN	42	58		37	63		19	81		38	62	
Patient delay												
Delayed*	31	69	0.00	37	72	0.06	14	86	0.20	30	70	0.01
Not delayed	46	55		37	63		19	81		43	57	
Health system delay												
Delayed^†^	37	63	0.88	31	69	0.89	18	82	0.11	36	34	0.63
Not delayed	36	64		32	68		13	87		64	67	

* Delay of >14 days from the onset of symptoms to the date of first contact with healthcare provider.

† Delay of >39 days between the first visit to a healthcare provider and diagnosis of a current disease.

PKR = Pakistani rupee; TBLN = tuberculous lymphadenitis; TBP = tuberculous pleuritis.

Table 3 presents a direct logistic regression analysis of four health domains to understand factors influencing patient QoL and the likelihood of experiencing severe or minor problems. Preliminary binary logistic regression confirmed the statistical significance of independent variables included in the model. Patients with TBP (odds ratio [OR] 1.640), a private salary (OR 1.681), longer diagnostic delays (OR 1.678), and living outside the city (OR 2.076) were more likely to have unfavourable effects on mobility. Private salary (OR 1.775) and out-of-city residence (OR 1.702) significantly impacted usual activities. Age 10–24 years and elementary education were predictors of reduced unfavourable effects on routine activities. For pain/discomfort, residing outside the city was significant, while age 10–24 years and private salary were predictors of reduced unfavourable effects. In the anxiety/depression domain, marriage and out-of-city residence had negative impacts.

**TABLE 3.** Logistic regression analysis predicting the possibility of unfavourable effects on QoL health domains at baseline (*n* =376).

QoL health domains	Factors	*P* value	OR (95% CI)
Mobility			
	TB pleuritis	0.03	1.64 (1.05–2.56)
	Income source: private sector salary	0.03	1.68 (1.05–2.70)
	Patient delay	0.03	1.68 (1.07–2.64)
	Rural place of residence	0.00	2.08 (1.34–3.22)
Usual activities			
	Age 10–24 years	0.01	0.50 (0.31–0.82)
	Basic elementary education	0.05	0.63 (0.39–1.00)
	Income source: private sector salary	0.03	1.78 (1.08–2.93)
	Rural place of residence	0.02	1.70 (1.08–2.70)
Pain/discomfort			
	Age 10–24 years	0.11	0.61 (0.33–1.13)
	Income source: private sector salary	0.12	1.62 (0.89–2.95)
	Rural place of residence	0.00	3.23 (1.77–5.90)
Anxiety/depression			
	Marital status	0.05	1.58 (1.00–2.49)
	Income source: private sector salary	0.13	1.45 (0.90–2.35)
	Patient delay	0.09	1.48 (0.94–2.34)
	Rural place of residence	0.00	2.18 (1.40–3.40)

QoL = quality of life; OR = odds ratio; CI = confidence interval.

Over a 2-year follow-up, eight (2%) more patients died, and 29 (8%) could not be contacted. Of 347 patients, 27 (8%) experienced symptom redevelopment after 2 years of treatment completion, with 22 suggesting EPTB. Two patients were retreated for recurrent EPTB; both were aged 10–24 years, previously treated for 6 months, and experienced 37–49 days of seeking initial diagnosis.

Univariate analysis findings in Figure 2B indicated that individuals over 45 years of age, those without baseline usual activity problems, and those without baseline anxiety or depression developed difficulties in these areas after 2 years of treatment completion.

## DISCUSSION

This study reveals that TBLN and TBP significantly impact patients' QoL across various physical and psychological health domains. PR-QoL improved substantially upon completion of ATT and remained stable after 2 years. However, 22 patients reported TB-related symptoms and reduced PR-QoL after 2 years, suggesting possible relapse. This is the first study to evaluate the impact of TBLN and TBP on PR-QoL among patients in Pakistan, including a 2-year post-treatment period.^10,12–14^

Both types of EPTB patients reported more physical and psychological health issues compared to previous studies, primarily on PTB. Before ATT initiation, nearly two-thirds reported extreme problems with mobility, usual activities, pain, discomfort, and anxiety or depression. This number is higher than that of previous studies on TB patients' QoL, including EPTB patients. A Pakistani study found over 50% of PTB patients had physical and psychological issues, suggesting EPTB affects health domains more than PTB.^15^

Pain or discomfort was the most common problem, reported by 84% of TBLN and TBP patients, a higher proportion than previous studies.^4,16–18^ However, comparing findings to prior studies is challenging, as most do not stratify results by disease site.

Financial status significantly impacts TB patients' QoL, with families earning over US$ 191.00per month reporting better QoL, consistent with studies in Pakistan, Thailand, and India.^19–21^ Chronic illnesses like TB pose a significant financial burden, leading to premature death and other challenges.^22^ In Pakistan, TB treatment is free in the public sector, but patients incur substantial out-of-pocket expenses for EPTB diagnosis, including travel costs and lost earnings.^23^ In this study, 66% of TBLN and TBP patients stopped working or reduced their capacity due to their illness, a higher proportion than in studies from India and Zanzibar.^9,11^

Anxiety and depression were more common in TBLN and TBP patients than in previous studies, with 65% reporting severe problems, highlighting the high risk of emotional health effects following a stigmatised illness diagnosis. Studies in Pakistan, India, and Malaysia show TB patients, especially those with PTB, are at higher risk of anxiety and depression before treatment.^13,24,25^ Timely diagnosis and basic treatment principles manage TBLN and TBP effectively, reducing fatality risk.

However, these patients are often ignored, leading to poor health-related QoL and lethal conditions. High TB burden countries, such as Pakistan, should provide psychological support for these patients to cope with emotional deprivation during diagnosis and treatment initiation.

Mobility domain problems were the least common but still higher (64%) than in other studies for all types of TB, including EPTB, in multiple countries.^12,16^ Respondents reported greater mobility-related problems due to reduced working capacity, TBLN site, and delays in seeking healthcare.

Over 90% of TBLN and TBP patients improved their PR-QoL after treatment, with positive impacts on mobility, pain, activities, and anxiety/depression. Studies in Yemen, Pakistan, Sri Lanka, and India showed similar results.^10,12,15,26^ However, patients who delayed seeking healthcare for over 14 days were more likely to have poor baseline PR-QoL.^11,27^ Additionally, 77–87% of patients with extended treatment reported baseline problems, suggesting timely diagnosis can prevent health problems and improve QoL. Healthcare providers and TB control programmes should address nonmedical aspects directly affecting EPTB patients.

Out-of-city residence was a common predictor for all four health domains, likely because geographic access to health facilities influences early TB diagnosis and treatment initiation.^28^

Older patients had a greater risk of unfavourable outcomes, possibly due to dependency on others to seek healthcare and higher comorbidity chances. Older patients may not exhibit typical TB symptoms, leading to a low index of suspicion by healthcare providers and poor QoL.

Pleural site infection is a significant predictor of negative mobility impact on QoL. PTB patients often delay seeking healthcare due to thoracentesis, an invasive procedure not available in all TB care facilities in Pakistan.^29^

Age >45 years is a significant risk factor for symptom recurrence in previously treated individuals, consistent with studies from South Korea, South Africa, and China,^30–32^ but not with a Pakistani study that found no significant association between advancing age and TB recurrence.^33^

EPTB patients without baseline problems performing routine tasks or psychological issues have a higher risk of developing PR-QoL problems after 2 years. This suggests that EPTB diagnosis disclosure and invasive therapy and drug treatment combinations significantly impact patients' physical and emotional health, persisting even after treatment.^34^

Disease-specific symptoms after 2 years of treatment without baseline psychological problems may result from anxiety and depression, causing poor treatment adherence and halting recovery. Emotional conflict in TB patients hinders recovery and causes relapses.^35^ Including emotional health services for pre-treatment, psychiatric assessment, and intervention is necessary for effective management.

The strength of this study is that it is the first assessment of changes in PR-QoL of TBLN and TBP patients, the most common forms of EPTB. However, the study had limitations, including not using generic tools like EQ-5D-5L, WHO QoL BREF, and Short-Form 36 (SF-36) and not being able to follow up with all enrolled patients face-to-face due to attrition. Additionally, only outpatients at a tertiary care-level hospital were enrolled, so results may not be generalisable to all EPTB patients.^9^

## CONCLUSION

EPTB, particularly TBLN and TBP, significantly impacts patients' physical and emotional health. Healthcare providers should address nonmedical aspects affecting patients' QoL, which may be incorporated into future national TB management guidelines and improve PR-QoL by ATT. Addressing these issues could reduce morbidity and improve QoL, potentially reducing mortality.
